# Effects of silent brain infarction on the hemorrhagic transformation and prognosis in patients with acute ischemic stroke after intravenous thrombolysis

**DOI:** 10.3389/fneur.2023.1147290

**Published:** 2023-05-11

**Authors:** Lulu Zhang, Shan Wang, Lanfeng Qiu, Juean Jiang, Jianhua Jiang, Yun Zhou, Dongxue Ding, Qi Fang

**Affiliations:** ^1^Department of Neurology, The First Affiliated Hospital of Soochow University, Suzhou, China; ^2^Department of Emergency, The First Affiliated Hospital of Soochow University, Suzhou, China; ^3^Department of General Medicine, The First Affiliated Hospital of Soochow University, Suzhou, China

**Keywords:** silent brain infarction, intravenous thrombolysis, acute ischemic stroke, hemorrhage transformation, functional outcomes

## Abstract

**Background:**

Silent brain infarction (SBI) is a special type of stroke with no definitive time of onset, which can be found on pre-thrombolysis imaging examination in some patients with acute ischemic stroke (AIS). However, the significance of SBI on intracranial hemorrhage transformation (HT) and clinical outcomes after intravenous thrombolysis therapy (IVT) is uncertain. We aimed to explore the effects of SBI on intracranial HT and the 3-month clinical outcome in patients with AIS after IVT.

**Methods:**

We consecutive collected patients who were diagnosed with ischemic stroke and received IVT from August 2016 to August 2022, and conducted a retrospective analysis in this study. The clinical and laboratory data were obtained from hospitalization data. Patients were divided into SBI and Non-SBI groups based on clinical and neuroimaging data. We use Cohen’s Kappa to assess the interrater reliability between the two evaluators, and multivariate logistic regression analysis was used to further assess the association between SBI, HT and clinical outcomes at 3 months after IVT.

**Results:**

Of the 541 patients, 231 (46.1%) had SBI, 49 (9.1%) had HT, 438 (81%) had favorable outcome, 361 (66.7%) had excellent outcome. There was no significant difference in the incidence of HT (8.2 vs. 9.7%, *p* = 0.560) and favorable outcome (78.4% vs. 82.9%, *p* = 0.183) between patients with SBI and Non-SBI. However, patients with SBI had a lower incidence of excellent outcome than the patients with Non-SBI (60.2% vs. 71.6%%, *p* = 0.005). After adjustment for major covariates, multivariate logistic regression analysis disclosed that SBI was independently associated with the increased risk of worse outcome (OR = 1.922, 95%CI: 1.229–3.006, *p* = 0.004).

**Conclusion:**

We found that SBI was no effect for HT after thrombolysis in ischemic stroke patients, and no effect on favorable functional outcome at 3 months. Nevertheless, SBI remained an independent risk factor for non-excellent functional outcomes at 3 months.

## Introduction

Silent brain infarction (SBI) refers to a patient who has no history of stroke or transient ischemic attacks (TIA), but cerebral infarction or encephalomalacia lesions are found on computed tomography (CT) or magnetic resonance imaging (MRI), with no corresponding symptoms and signs of neurological impairment (1). Compared with symptomatic cerebral infarction, the lesions of SBI are usually relatively smaller in size and may have undergone a chronic ischemic preconditioning process, contributing to the absence of clinical symptoms of SBI (2). Previous reports have established that SBI is prevalent in both healthy older adults as well as in specific populations, such as those with hypertension, diabetes, atrial fibrillation, and other conditions (3). Imaging examination, especially MRI is indispensable for the diagnosis of SBI. Recent studies have suggested that MRI examination for the diagnosis of SBI should include at least T1-weighted imaging (T1WI) and T2-weighted imaging (T2WI) sequences. In this regard, SBI with an infarct diameter of ≥3 mm, were defined as hypointense lesions on T1WI, while on T2WI they were characterized as hyperintense (4).

In acute ischemic stroke (AIS) patients without intravenous thrombolysis therapy (IVT), studies have shown that SBI was independently associated with lower stroke severity at admission and good function outcome at discharge (5). Another retrospective study of 115 patients with first-ever ischemic stroke without advanced leukoaraiosis found that patients with multiple SBI had severer neurological and had larger infarcts in ischemic stroke than those without SBI, these patients also did not receive intravenous thrombolysis (6). Currently, to our knowledge, no retrospective study has probed the effect of SBI on HT and clinical outcome in patients with acute ischemic stroke after IVT. Therefore, the effect of SBI on HT and the clinical outcome after IVT in patients with AIS needs to be further clarified.

In this study, we retrospectively analyzed 541 patients with first-ever ischemic stroke who underwent intravenous thrombolysis therapy. In particular, we examined the HT and clinical outcome after intravenous thrombolysis therapy in the SBI and Non-SBI groups. We further determined the influence of SBI on the risk of worse outcome for patients with AIS following intravenous thrombolysis therapy.

## Subjects and methods

### Patients

**Figure 1:**
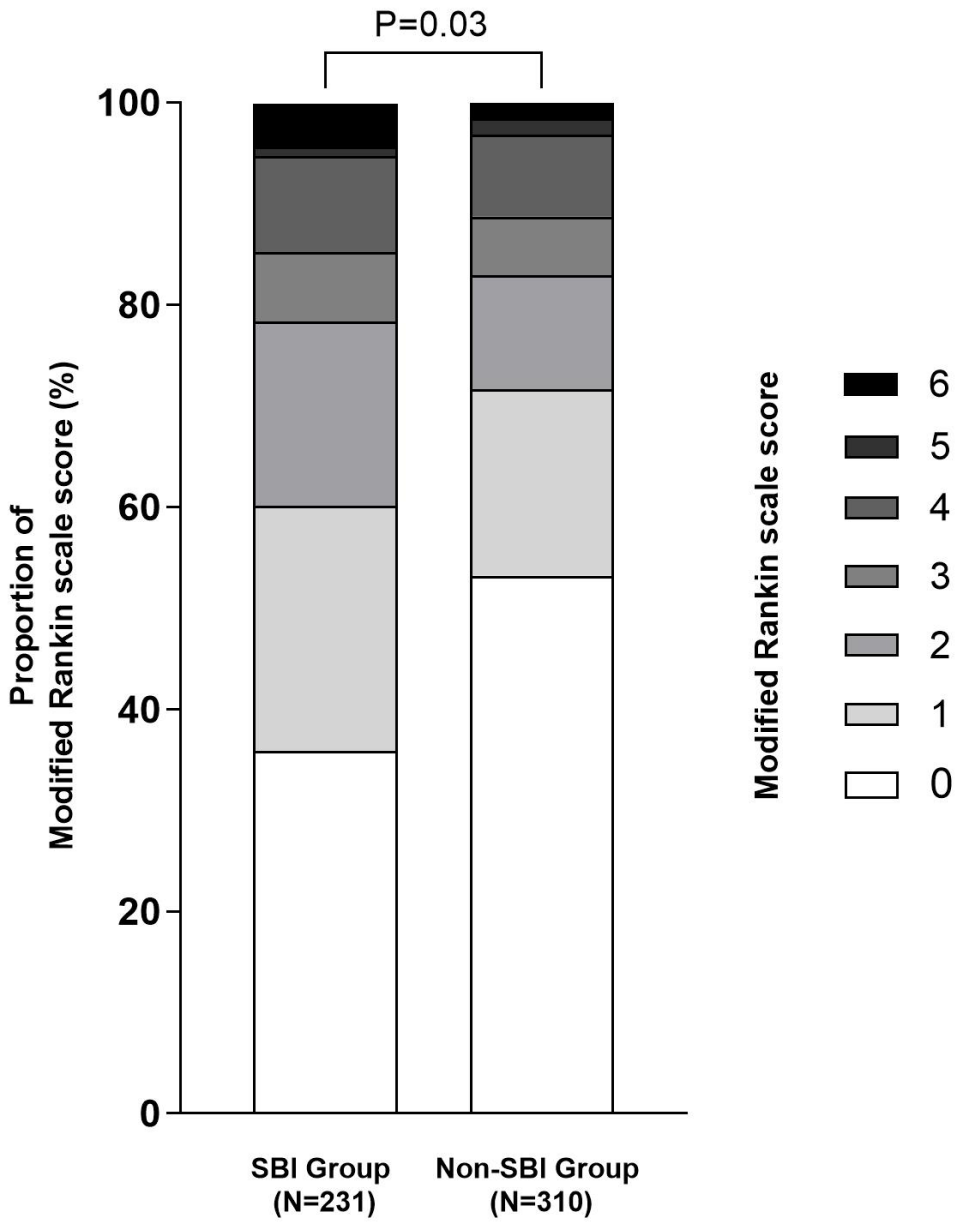
The flow chart of the study.

Totally 909 patients who suffered from an ischemic stroke within 4.5 h of onset and received recombinant tissue plasminogen activator (rt-PA) thrombolytic therapy in the emergency green channel of the First Affiliated Hospital of Soochow University between August 2016 and August 2022 were enrolled in this study. Eligible patients were further included if they had: (1) no history of stroke or TIA, (2) completed intravenous thrombolysis therapy, (3) completed a cranial CT scan within 24 h after intravenous thrombolysis therapy, or (4) completed head MRI examination within 1 week. On the other hand, patients were excluded if they had: (1) history of stroke or TIA, (2) not completed intravenous thrombolysis therapy, (3) intravenous thrombolysis combined with thrombectomy, (4) lacking imaging data，or (5) lacking laboratory data. The flow chart of the study is shown in Figure 1. Finally, 541 patients were assigned to the SBI and Non-SBI groups. This study was approved by the Ethics Committee of the First Affiliated Hospital of Soochow University (2020 No.267). In accordance with national legislation and institutional requirements, written informed consent is not required for this study.

### Intravenous rt-PA thrombolysis therapy

The standard dosage was 0.9 mg per kilogram of body weight (10% as a bolus for 1 min and remaining 90% as an infusion for 60 min; maximum dose, 90 mg). The low dosage was 0.6 mg per kilogram of body weight (15% as a bolus for 1 min and remaining 85% as an infusion for 60 min; maximum dose, 60 mg). All patients received only one dose.

### Clinical and laboratory data

We recorded the following baseline information of patients: gender, age, NIHSS score on admission, systolic and diastolic blood pressure on admission, head CT and MRI imaging examinations, and TOAST classification. We also collected previous medical history of patients including hypertension, diabetes mellitus, hyperlipidemia, atrial fibrillation, smoking and drinking history, and antithrombotic medication history (antiplatelet agents or any type of oral anticoagulants). Consequently, we estimated the 3-month outcome using the modified Rankin Scale (mRS). We defined mRS scores ≥3 as poor outcome and mRS scores ≤2 as favorable outcome, mRS scores ≥2 as worse outcome and mRS scores 0 or 1 as excellent outcome. The 3-month follow-up data was obtained by trained nurse through outpatient visits or telephone contact with patients or relatives.

### Imaging analysis

MRI data were acquired using a clinical 3.0 Tesla MR scanner, whereas follow-up CT imaging was performed using a clinical 64 slice CT scanner. Specifically, structural MRI comprised of transversal diffusion-weighted imaging (DWI), fluid-attenuated inversion recovery sequence (FLAIR), T1WI, and T2WI. Silent brain infarction on MRI lesions ≥3 mm, were characterized as hypointense on T1WI and hyperintense on T2WI (4). All MRIs were reviewed separately by two experienced neuroradiologists who were blinded to the identity of patients and their clinical information.

### Hemorrhage transformation and symptomatic intracranial hemorrhage

Following 24 h thrombolysis, all enrolled patients underwent head CT examination to evaluate whether there was HT. HT was defined as no manifestation of bleeding on head CT at admission, but found on CT or MRI at follow-up within 7 days after intravenous thrombolysis (7). According to ECASSIII (8), symptomatic intracranial hemorrhage (sICH) was defined as any apparent HT that was associated with clinical deterioration (≥4 points in the NIHSS score), or that led to death and that was identified as the predominant cause of the neurologic deterioration. Notably, HT was examined during follow-up imaging by a neurologist and radiologist.

### Statistical analysis

Kolmogorov–Smirnov test was used to test normal distribution of the continuous variables. Normally distributed data were presented as mean and standard deviation (SD) and Non-normally distributed data as median with interquartile range (IQR) or counts and percentages. And categorical variables are presented as frequencies and percentages. Moreover, we used student’s *t*-test or Mann–Whitney U test for continuous variables according to the normality and *χ*^2^ test or Fisher exact test for categorical variables. The two-tailed *p*-values of <0.05 were considered statistically significant. We used univariate analysis to compare the differences in baseline data between the SBI and non-SBI group. The results showed a statistically significant difference on clinical outcome. To further explore the effect of SBI on clinical outcome after intravenous thrombolysis, we first used univariate analysis to compare baseline data between the excellent outcome group and the worse outcome group. Then, we adjusted for age, sex, and variables with *p* < 0.1 [old age, hypertension, atrial fibrillation history, anti-thrombotic history, admission NIHSS score, TOAST subtypes, glucose level, admission lymphocyte count, admission neutrophil count, admission platelet count, international normalized ratio, homocysteine level and SBI] in univariate analysis as major covariates into the multivariate logistic regression model. The relationship between SBI and clinical outcomes was assessed by multivariate logistic regression analysis. The interrater reliability between the two observers was assessed based on Cohen’s Kappa for SBI and HT presence. A Cohen’s Kappa (*k*) of ≤0.1 corresponds to no agreement, 0.1 < *k* ≤ 0.4 weak agreement, 0.4 < *k* ≤ 0.6 good agreement, 0.6 < *k* ≤ 0.8 strong agreement and 0.8 < *k* ≤ 1 complete agreement. Statistical analyses were performed using the statistical package for social sciences, version 22.0 (IBM SPSS Statistics, Armonk, NY, United States).

## Results

### Baseline characteristics of patients

A total of 909 patients with AIS who received IVT treatment were screened for eligibility. Of these patients, 172 patients were first excluded because of a previous history of stroke or TIA. 100 patients did not receive cranial MR examination after thrombolysis, and 91 patients received incomplete laboratory data, 5 patients were lost for follow-up. Finally, 541 patients were included in our analysis (Figure 1), and the overall characteristics of participants are shown in Table 1. The median age was 66 (57–75) years and 323 (59.7%) were males. The median NIHSS score on admission was 5 (3–10). The median time to treat was 177.0 min (136.5–215.0). Of these patients, 231 (42.7%) patients had SBI lesions (*k* = 0.893, 95%CI: 0.855–0.929, *p* = 0.000), 49 (9.1%) patients experienced HT (*k* = 1.000, 95%CI: 1.000–1.000, *p* = 0.000), which 18 (3.3%) had sICH (*k* = 1.000, 95%CI: 1.000–1.000, *p* = 0.000). Results for the interrater reliability correspond to a complete agreement. There were 438 (81%) patients with MRS 0–2 and 361 (66.7%) MRS 0–1 at 90 days.

**Table 1:** Characteristics of the patients at baseline between SBI group and Non-SBI group.

	Total patients (*n* = 541)	SBI group (*n* = 231)	Non-SBI group (*n* = 310)	*p* value
Demographics				
Age, (years)	66 (57–75)	69 (69–76)	65 (54–74)	0.000
Male, *n* (%)	323 (59.7%)	107 (67.9%)	216 (69.7%)	0.951
Cardiovascular risk factors, *n* (%)				
Hypertension	378 (69.9%)	178 (77.1%)	200 (64.5%)	0.020
Diabetes	140 (25.9%)	70 (30.3%)	70 (22.6%)	0.040
Atrial fibrillation	107 (19.8%)	50 (21.6%)	57 (18.4%)	0.347
Smoke	154 (28.5%)	77 (33.3%)	77 (24.8%)	0.030
Drink	109 (20.1%)	55 (23.8)	54 (17.4)	0.067
Hyperlipemia	33 (6.1%)	12 (5.2%)	21 (6.8%)	0.448
Medication history, *n* (%)				
Anti-thrombotic	25 (4.6%)	9 (3.9%)	16 (5.2%)	0.488
Physiological data on admission				
SBP (mmHg)	157.4 ± 24.31	159.3 ± 24.88	155.96 ± 23.675	0.113
DBP (mmHg)	88.0 (79.0–98.0)	88.0 (78.0–98.0)	88.0 (79.0–98.3)	0.698
Baseline NIHSS scores	5 (3–10)	5 (3–10)	5 (3–10)	0.893
OTT time	177.0 (136.5–215.0)	175 (135.0–211.0)	177.5 (138.5–215.0)	0.838
Standard-dose rt-PA	511 (94.5%)	218 (94.4%)	293 (94.5%)	0.942
TOAST classification, *n* (%)				0.008
LAA	232 (42.9%)	100 (43.3%)	132 (42.6%)	
CE	96 (17.7%)	44 (19.0%)	52 (16.8%)	
SAA	142 (26.2%)	70 (30.3%)	72 (23.2%)	
SOE	19 (3.5%)	6 (2.6%)	13 (4.2%)	
SUE	52 (9.6%)	11 (4.8%)	41 (13.2%)	
Laboratory test data				
GLU (mmol/L)	6.92 (5.77–8.73)	6.88 (5.66–9.19)	6.925 (5.86–8.45)	0.962
WBC (×10^9^/L)	7.50 (6.38–9.28)	7.52 (6.48–9.26)	7.49 (6.29–9.37)	0.352
LY (×10^9^/L)	1.65 (1.19,2.2445)	1.61 (1.17,2.19)	1.72 (1.22,2.27)	0.390
NE (×10^9^/L)	4.98 (3.86,6.85)	4.98 (4.05,6.87)	4.97 (3.79,6.7375)	0.436
PLT (×10^9^/L)	197.0 (162.5–240.0)	191.0 (159.0–234.0)	201.0 (168.0–242.3)	0.670
LDL-C (mmol/L)	2.81 ± 0.93	2.75 ± 0.85	2.84 ± 0.98	0.261
CHO (mmol/L)	4.45 (3.85–5.16)	4.40 (3.79–5.21)	4.50 (3.88–5.11)	0.431
UA (μmol/L)	303.6(242.8–363.8)	307.9 (247.0–371.1)	301.4 (239.6–361.6)	0.235
Cr (μmol/L)	68 (58–79)	69 (59–81)	67 (58–78)	0.085
INR	1.02 (0.97–1.08)	1.02 (0.97–1.08)	1.02 (0.97–1.08)	0.791
APTT (s)	32.0 (27.9–35.2)	31.5 (26.6–35.0)	32.3 (28.6–35.4)	0.034
FIG (g/L)	3.06 (2.62–3.58)	3.06 (2.61–3.61)	3.05 (2.62–3.55)	0.657
PT (s)	13.0 (12.3–13.6)	12.9 (12.2–13.7)	13.0 (12.4–13.53)	0.268
Hcy	10.7 (8.8–13.6)	11.4 (9.4–14.8)	10.4 (8.4–13.1)	0.001
Outcome, *n* (%)				
HT	49 (9.1%)	19 (8.2%)	30 (9.7%)	0.560
SICH	18 (3.3%)	11 (4.8%)	7 (2.3%)	0.108
mRS (0–2)	438 (81%)	181 (78.4%)	257 (82.9%)	0.183
mRS (0–1)	361 (66.7%)	139 (60.2%)	222 (71.6%)	0.005

Data are expressed as mean ± standard deviation (SD), median with interquartile range (IQR) or percentage. SBP, systolic blood pressure; DBP, diastolic blood pressure; NIHSS, the national institutes of health stroke scale; TOAST, the trial of org 10,172 in acute stroke treatment; LAA, large-artery atherosclerosis; SAA, small-artery occlusion; CE, cardioembolism; SOE, stroke of other determined cause; SUE: stroke of undetermined cause; GLU, glucose; WBC: white blood cell; LY, lymphocyte; NE, neutrophil; PLT, platelet; LDL-C, low-density lipoprotein cholesterin; CHO, total cholesterol; UA, uric acid; Cr, creatinine; INR, international normalized ratio; APTT, activated partial thromboplastin time; FIG, fibrinogen; PT, prothrombin time; HCY, homocysteine; HT, hemorrhagic transformation; sICH, symptomatic intracranial hemorrhage; SBI, silent brain infarction.

### Characteristics between patients with and without SBI

**Figure 2:**
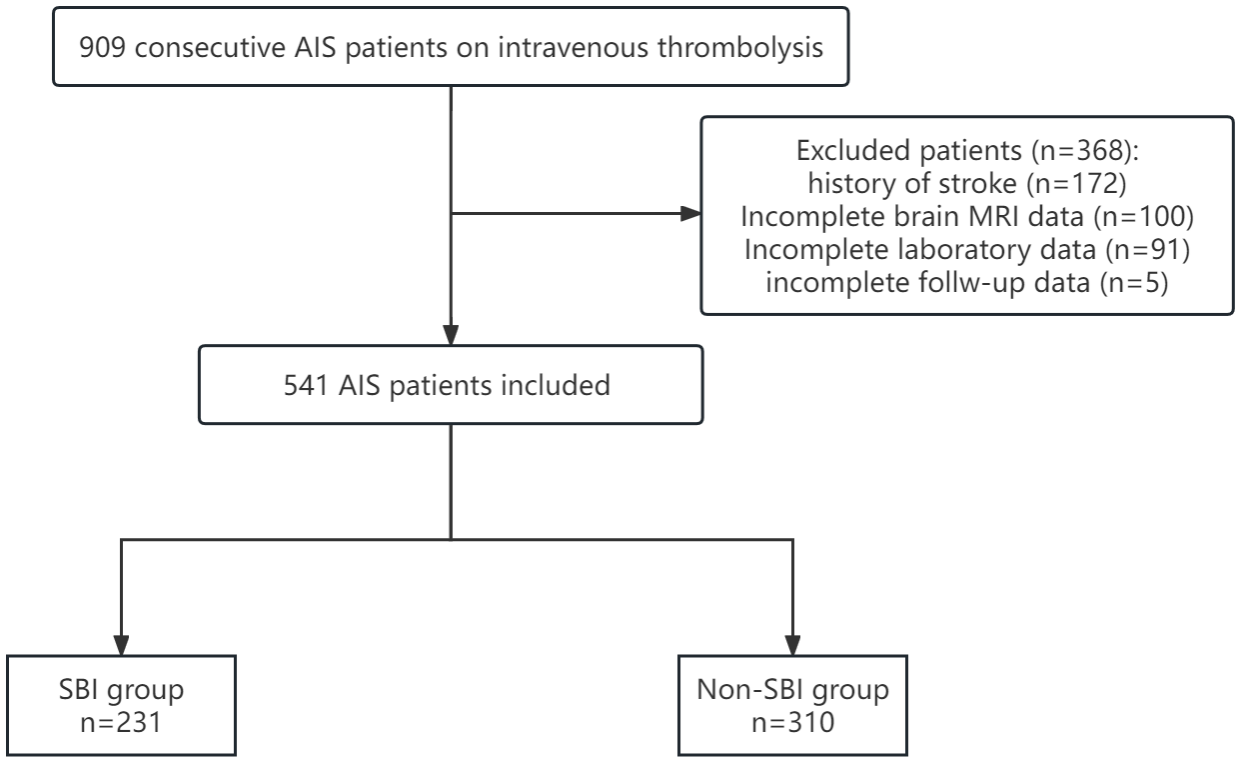
Distribution of the total mRS scores as stratified by SBI groups.

Table 1 shows the baseline characteristics of SBI group and Non-SBI group. 231 (42.7%) patients were classified as SBI group, whereas 310 (57.3%) patients were assigned to the Non-SBI group. Patients with SBI were usually older and consisted more of hypertension, diabetes and smoking history than patients without SBI [69 (69–76) vs. 65 (54–74), *p* = 0.000; 178 (77.1%) vs. 200 (64.5%), *p* = 0.020; 70 (30.3%) vs. 70 (22.6%), *p* = 0.040; 77 (33.3%) vs. 77 (24.8%), *p* = 0.030]. The TOAST subtype distribution also showed statistically significant differences between the two groups (*p* = 0.008). Furthermore, patients with SBI had higher serum homocysteine levels and shorter activated partial thromboplastin time than those without SBI [11.4 (9.4–14.8) vs. 10.35 (8.4–13.1), *p* = 0.001；31.5 (26.6–35.0) vs. 32.3 (28.6–35.4), *p* = 0.034. Table 1]. For clinical outcome, there was no significant difference in the incidence of HT [19 (8.2%) vs. 30 (9.7%), *p* = 0.560] and sICH [11 (4.8%) vs. 7 (2.3%), *p* = 0.108] between the two groups. Although there was no significant difference in the incidence of favorable outcome between the two groups [181 (78.4%) vs. 257 (82.9%), *p* = 0.183], the incidence of excellent outcome was lower in the SBI group than the Non-SBI group [139 (60.2%) vs. 222 (71.6%), *p* = 0.005, Figure 2].

### Relationship between SBI and 3-month excellent outcomes

Table 2 shows the baseline characteristics of the excellent function outcome group versus the worse function outcome group. Univariate analysis showed that age, hypertension, atrial fibrillation, antiplatelet medication history, TOAST classification, and admission NIHSS score were significant differences between two groups (*p* < 0.05). In the laboratory test results, glucose level, admission neutrophil and lymphocyte count, serum HDL-C level, serum TG level, serum HCY level, and International Normalized Ratio also significant differences (*p* < 0.05). The rate of SBI in patients with worse function outcome was higher than that in patients with excellent function outcome (*p* = 0.005).

**Table 2:** Univariate analysis of baseline factors associated with clinical outcome.

	mRS 0–1 (*n* = 361)	mRS 2–6 (*n* = 180)	*p* value
Demographics			
Age, (years)	65 (55–73)	71.5 (62–79)	0.000
Male, *n* (%)	256 (70.9)	120 (66.7)	0.312
Cardiovascular risk factors, *n* (%)			
Hypertension	240 (66.5)	138 (76.7)	0.015
Diabetes	86 (23.8)	54 (30)	0.122
Atrial fibrillation	58 (16.1)	49 (27.2)	0.020
Smoke	103 (28.5)	51 (28.3)	0.962
Drink	78 (21.6)	31 (17.2)	0.231
Hyperlipemia	23 (6.4)	10 (5.6)	0.709
Medication history, *n* (%)			
Anti-thrombotic	12 (3.3)	13 (7.2)	0.042
Physiological data on admission			
SBP (mmHg)	156.7 ± 24.067	158.77 ± 24.564	0.855
DBP (mmHg)	88 (79–99)	88 (78–98)	0.633
Baseline NIHSS scores	4 (1–7)	10 (6–15)	0.000
OTT time	176 (140–215)	179.5 (135–210.75)	0.835
Standard-dose rt-PA (%)	343 (95)	168 (93.3)	0.421
TOAST classification, *n* (%)			0.000
LAA	142 (39.3)	90 (50.0)	
CE	52 (14.4)	44 (24.4)	
SAA	111 (30.7)	31 (17.2)	
SOE	15 (4.2)	4 (2.2)	
SUE	41 (11.4)	11 (6.1)	
Laboratory test data			
GLU (mmol/L)	6.73 (5.66–8.36)	7.35 (6.00–10.10)	0.001
WBC (×109/L)	7.48 (6.245–9.175)	7.59 (6.515–9.625)	0.381
LY (×109/L)	1.7 (1.250–2.335)	1.455 (1.0225–1.9275)	0.000
NE (×109/L)	4.74 (3.705–6.660)	5.355 (4.1625–7.2250)	0.006
PLT (×109/L)	201.00 (167.00–241.50)	187.5 (154.75–233.75)	0.038
LDL-C (mmol/L)	2.8136 ± 0.948	2.7884 ± 0.893	0.497
CHO (mmol/L)	4.43 (3.830–5.175)	4.455 (3.91–5.085)	0.577
UA	301.3 (244.8–361.85)	307.45 (238.325–375.875)	0.454
CR (μmol/L)	67.50 (58.00–77.75)	68.00 (58.00–83.725)	0.268
INR	1.01 (0.97–1.08)	1.04 (0.98–1.09)	0.026
APTT (S)	32.0 (28.35–35.3)	32.05 (27.00–35.00)	0.410
FIG (g/L)	3.03 (2.60–3.55)	3.085 (2.6425–3.71)	0.294
PT (S)	12.9 (12.3–13.5)	13.1 (12.3–13.7)	0.159
HCY	10.5 (8.6–13.15)	11.25 (9.3–14.9)	0.025
SBI	139 (38.5)	92 (51.1)	0.005

Data are expressed as mean ± standard deviation (SD), median with interquartile range (IQR) or percentage. SBP, systolic blood pressure; DBP, diastolic blood pressure; NIHSS, the national institutes of health stroke scale; TOAST, the trial of org 10,172 in acute stroke treatment; LAA, large-artery atherosclerosis; SAA, Small-artery occlusion; CE, cardioembolism; SOE, stroke of other determined cause; SUE, stroke of undetermined cause; GLU, glucose; WBC, white blood cell; LY, lymphocyte; NE, neutrophil; PLT, platelet; LDL-C, low-density lipoprotein cholesterin; CHO, total cholesterol; UA, uric acid; Cr, creatinine; INR, international normalized ratio; APTT, activated partial thromboplastin time; FIG, fibrinogen; PT, prothrombin time; HCY, homocysteine; SBI, silent brain infarction.

### Univariable and multivariable analyses of 3-month worse outcomes

In univariable analyses, old age, hypertension and atrial fibrillation history, anti-thrombotic history, admission NIHSS score, TOAST subtypes, glucose level, admission lymphocyte count, admission neutrophil count, admission platelet count, international normalized ratio, homocysteine level and SBI were associated with worse outcome in AIS after rt-PA treatment. After age and sex adjustment, initial NIHSS, TOAST subtypes, glucose level, admission lymphocyte count, admission neutrophil count and SBI were significantly associated with worse outcome at 3 months after discharge. To figure out whether SBI were an independent prognostic indicator for worse outcome in 3 months. Variables with *p* < 0.1 in the univariate analysis and the Age- and sex-adjusted analysis were included in the multivariate logistic regression model. After age, sex, and multivariate adjustment, only initial NIHSS (OR = 1.239, 95% CI: 1.180–1.302, *p* = 0.000), GLU (OR = 1.079, 95%CI: 1.019–1.144, *p* = 0.010), and admission lymphocyte count (OR = 0.658, 95%CI: 0.498–0.869, *p* = 0.003), SBI (OR = 1.922, 95%CI: 1.229–3.006, *p* = 0.004) were significantly related to worse outcomes at 3 months after discharge (Table 3).

**Table 3:** Multivariate logistic regression analysis for worse outcomes predictors at 3 months (mRS score 2–6).

Variables	Age-and sex-adjusted		Multivariate adjusted	
	OR (95%CI)	*p* value	OR (95%CI)	*p* value
Hypertension	1.417 (0.925–2.170)	0.109	1.143 (0.690–1.892)	0.604
Atrial fibrillation	1.296 (0.815–2.060)	0.273	1.062 (0.400–2.820)	0.904
Anti-thrombotic	1.763 (0.774–4.014)	0.177	2.099 (0.759–5.810)	0.153
NIHSS	1.215 (1.164–1.269)	0.000	1.239 (1.180–1.302)	0.000
TOAST classification				
LAA	–	–	–	–
CE	1.101 (0.669–1.813)	0.704	0.493 (0.175–1.392)	0.182
SAA	0.535 (0.329–0.870)	0.012	0.622 (0.356–1.086)	0.095
SOE	0.556 (0.174–1.782)	0.323	0.736 (0.189–2.865)	0.659
SUE	0.490 (0.234–1.023)	0.058	0.741 (0.327–1.679)	0.473
GLU	1.058 (1.006–1.111)	0.027	1.079 (1.019–1.144)	0.010
LY	0.683 (0.540–0.864)	0.001	0.658 (0.498–0.869)	0.003
NE	1.098 (1.025–1.176)	0.008	0.976 (0.895–1.065)	0.590
PLT	1.000 (0.997–1.002)	0.747	1.000 (0.999–1.002)	0.919
INR	0.806 (0.466–1.395)	0.441	0.496 (0.047–5.248)	0.560
HCY	1.006 (0.993–1.019)	0.366	1.009 (0.989–1.030)	0.371
SBI	1.479 (1.020–2.146)	0.039	1.922 (1.229–3.006)	0.004

NIHSS, the national institutes of health stroke scale; TOAST, the trial of org 10,172 in acute stroke treatment; LAA, large-artery atherosclerosis; SAA, small-artery occlusion; CE, cardioembolism; SOE, stroke of other determined cause; SUE, stroke of undetermined cause; GLU, glucose; LY, lymphocyte; NE, neutrophil; PLT, platelet; INR, international normalized ratio; HCY, homocysteine; SBI, silent brain infarction.

## Discussion

To our knowledge, this is the first research on the effect of SBI on hemorrhagic transformation and clinical outcomes after thrombolysis in patients with acute ischemic stroke. We found that SBI was relatively common in patients with a first-ever ischemic stroke, and SBI was an independent risk factor for worse outcome (OR = 1.922, 95%CI: 1.229–3.006, *p* = 0.004), but not an independent risk factor for HT and favorable outcome after IVT.

Silent brain infarction (SBI) refers to lesions discovered via neuroimaging that lack associated clinical symptomology (9). The prevalence of SBI is about 10–20% in the general population, with an annual incidence rate of 2 to 4% (10). Previous studies have found that SBI is present in about 33.4% of patients with a first-ever ischemic stroke (11). Risk factors for SBI are consistent with ischemic stroke, such as advanced age, hypertension, and diabetes (3). Kim et al. found that hyperhomocysteinemia is an independent risk factor (OR = 4.78; 95%CI: 2.45–9.33) for SBI (12). Wang and colleagues found that the higher prevalence of SBI was associated with incompleteness of circle of Willis in patients with internal carotid artery stenosis (13). Ito et al. indicated that aortic stenosis was associated with a high prevalence of SBI, and the CHA_2_DS_2_-VASc score (≥4) and eGFR (<60 mL/min/1.73m^2^) are useful for risk stratification (14). Nacafaliyev reported that patients with moderate and severe sleep apnea syndrome were at higher risk of developing SBI and noted that desaturations during sleep may affect infarct formation (15). In the baseline data of this study, we found that not only were TOAST subtype distributions different between the two groups, but also that patients with SBI had shorter activated partial thromboplastin times than those without SBI.

Studies have shown that SBI is not completely asymptomatic, and oldish people with SBI have an increased risk of dementia and a faster decline in cognitive function than those without such lesions (16, 17). In addition, a prospective study concluded that overt and silent brain infarction had similar effects on cognitive decline (18). A meta-analysis of 14,764 subjects with a mean follow-up time of 25.7 to 174 months uncovered that about 20% of the stroke-free older adults had SBI, and indicated that the probability of SBI patients developing symptomatic cerebral infarction was twice as high as that of healthy people (1). Carotid endarterectomy is one of the treatments to reduce the risk of stroke in patients with asymptomatic carotid stenosis, the study found that the presence of SBI was independently associated with a higher risk of postoperative stroke for carotid endarterectomy (19). Multiple SBIs have more severe neurological deficits and larger infarcts for ischemic stroke those without no SBI in patients with first-ever ischemic stroke without advanced leukoaraiosis (6). Another community-based study showed that silent infarcts did not appear to affect the prognosis of stroke (20).

Admission glucose level and neutrophil-to-lymphocyte ratio can predict 3-month functional outcome in AIS patients (21, 22). Our data also indicated that patients with high admission glucose levels and lymphocyte were more likely to have worse outcome at 3 months. Another retrospective study included 981 ischemic stroke patients and found that recent clinically silent infarcts (RSIs) was not associated with a worse clinical outcome in AIS patients with IVT. Although clinical outcome were measured by the mRS at discharge, their conclusions argue against RSIs as a contraindication for IVT (23). A small sample study without thrombolysis found lower 3-month mRS Scores and better clinical outcomes in the SBI present group compared to the SBI absent group (24). In this study, we evaluated mRS Scores at 3 months to explore the effect of SBI on clinical outcomes after IVT. The results showed that a similar number of patients achieved favorable outcomes in the Non-SBI group compared to the SBI group, Non-SBI group was slightly superior over SBI group in terms of likelihood of achieving an excellent outcome.

When symptomatic cerebral infarction occurs, intravenous thrombolytic therapy with rt-PA within 4.5 h can effectively improve the clinical prognosis of patients after the removal of thrombolytic contraindications (25). Hemorrhagic transformation remains a primary adverse reaction of intravenous thrombolytic therapy after acute cerebral infarction, which is closely associated with the clinical outcome of patients. Previous studies have found that older age, higher diastolic blood pressure, NIHSS score ≥ 13, OTT ≥ 180 min, etc., are potential risk factors for HT after intravenous thrombolysis (26, 27). Pretreatment MRI can identify recent silent cerebral infarction (RSCI) and two small sample studies suggest that RSCI does not increase the risk of hemorrhagic transformation after intravenous thrombolysis in patients with acute cerebral infarction (28, 29). Although RSCI can be identified by the use of MRI, the onset to treatment time of AIS patients was often prolonged by MRI examination before thrombolytic. Therefore, we systematically reviewed the whole group of AIS patients who completed MRI examination after intravenous thrombolytic, and disclosed the incidence of SBI confirmed by MRI in the entire cohort and its influence on hemorrhagic transformation after intravenous thrombolysis. Our results were similar to the findings of the two retrospective studies described above, and SBI, like RSCI, did not increase the risk of hemorrhagic transformation after intravenous thrombolysis.

However, there are some limitations in this study. Firstly, the present study was a retrospective analysis with limited sample size. Second, the findings of this study are limited by selection bias, as patients who did not complete a cranial MRI examination were not included in the MRI-based study, and these excluded patients may be associated with more severe intracranial hemorrhage and poorer clinical outcomes. Third, acute large cerebral infarction occurred in lobes with previous small SBI lesions in the cortex or medulla of these lobes. SBI lesions may be compressed by edema in acute cerebral infarction and cannot be detected by MRI. This hypothesis has not been proven, but it could lead to information bias. Last, we did not further explore the relationship between SBI and post-stroke cognitive impairment and post-stroke depression. Further multi-faceted exploration should be carried out in multi-center study with a large sample size.

## Conclusion

We found that SBI was no effect for HT after thrombolysis in ischemic stroke patients, and no effect on favorable functional outcome at 3 months. Nevertheless, SBI remained an independent risk factor for non-excellent functional outcomes at 3 months.

## Data availability statement

The raw data supporting the conclusions of this article will be made available by the authors, without undue reservation.

## Ethics statement

The studies involving human participants were reviewed and approved by the Ethics Committee of the First Affiliated Hospital of Soochow University. The ethics committee waived the requirement of written informed consent for participation. No potentially identifiable human images or data is presented in this study.

## Author contributions

YZ, DD, and QF contributed to conception and design of the study. LZ wrote the first draft of the manuscript. SW organized the database. QF contributed the patient follow-up. JiJ and JuJ performed the statistical analysis. YZ and DD revised the manuscript. All authors contributed to manuscript revision, read, and approved the submitted version.

## Funding

This work was supported by the National Key Research and Development Program of China (2017YFE0103700), the National Natural Science Foundation of China (No. 82001219 to DD), the Natural Science Foundation of Jiangsu Province (BK20190183), and the Advanced Research Project of Suzhou University of Natural Science Foundation (SDY2012B25).

## Conflict of interest

The authors declare that the research was conducted in the absence of any commercial or financial relationships that could be construed as a potential conflict of interest.

## Publisher’s note

All claims expressed in this article are solely those of the authors and do not necessarily represent those of their affiliated organizations, or those of the publisher, the editors and the reviewers. Any product that may be evaluated in this article, or claim that may be made by its manufacturer, is not guaranteed or endorsed by the publisher.
